# Breast cancer-preventive behaviors: exploring Iranian women’s experiences

**DOI:** 10.1186/1472-6874-14-41

**Published:** 2014-03-10

**Authors:** Maryam Khazaee-Pool, Ali Montazeri, Fereshteh Majlessi, Abbas Rahimi Foroushani, Saharnaz Nedjat, Davoud Shojaeizadeh

**Affiliations:** 1 Department of Health Education and Promotion, School of Public Health, Tehran University of Medical Sciences, P.O. Box 15875–6951, Tehran, Iran; 2 Mental Health Research Group, Health Metrics Research Center, Institute for Health Sciences Research, ACECR, P.O. Box 13185–1488, Tehran, Iran; 3 Department of Epidemiology and Biostatistics, School of Public Health, Tehran University of Medical Sciences, Tehran, Iran

**Keywords:** Preventive behaviors, Breast cancer, Iranian women, Qualitative study

## Abstract

**Background:**

Breast cancer-preventive behaviors are critical for community and women’s health. Although many studies have addressed women’s knowledge and attitudes toward breast cancer, little information is available about their experiences of breast cancer preventive behaviors. This study aimed to explore the experiences of Iranian women regarding preventive behaviors.

**Methods:**

This was a qualitative study. A sample of Iranian women aged 30 years and over was selected purposefully. Data collected through focus group and semi-structured audiotaped interviews and were analyzed by conventional content analysis.

**Results:**

The following five main themes emerged from the analysis: attitude toward breast cancer and preventive behaviors, stress management, healthy lifestyle, perceived social support and individual/environmental barriers. The findings showed that women were highly motivated to preventive behaviors of breast cancer but faced considerable challenges.

**Conclusions:**

The findings indicated that increased awareness, positive attitudes, stronger motivational factors, and fewer barriers toward preventive behaviors are most important parameters that might encourage women to practice breast cancer-preventive behaviors.

## Background

Breast cancer is the most common cancer among women and is the leading cause of female deaths due to cancer [1]. In Iran, breast cancer is amongst the five most common cancers and ranks first among cancers diagnosed in women. Studies from Iran suggest that breast cancer affects Iranian women at least one decade younger than women in developed countries, with the mean age ranging from 47.1 to 48.8 years [2]. Although the incidence of breast cancer in Iran is still relatively low (about 23 per 100,000), the number of patients with newly diagnosed breast cancer is increasing [3].

There are several risk factors for breast cancer and are numerous, but some life style and behavioral factors may have an important role on increased risk for developing breast cancer including dietary habits [4,5], low physical activity [6,7], smoking [8], alcohol consumption [9,4] and experiencing high level of stress [10]. These lifestyle factors are not only important risk factors for developing breast cancer, but also impact health outcomes and quality of life. Thus it is argued that the most beneficial and effective means of reducing breast cancer burden, and mortality is prevention and early detection [11].

It has been suggested that the appropriate method for breast cancer prevention and early detection is performing preventive behaviors and these might include lifestyle modification and screening behaviors (mammography, clinical breast examination and breast self-examination).

Lifestyle modification can help to reduce risk of breast cancer to a large scale [1]. Previous studies demonstrated that lifestyle interventions such as changing dietary habits [12,13], performing adequate physical activity [14-18], quiet smoking and reducing alcohol drinking [19,20] may prevent breast cancer and decrease the risk. Also, Magné et al. stated that there are positive relationship between breast cancer prevention and healthy lifestyle since for instance physical activity can prevent excessive weight gain and thus reduce risk of breast cancer [21].

Screening behaviors including mammography, clinical breast examination and breast self-examination are also considered to be effective for early detection and reduced mortality due to breast cancer [22-24], although guidelines for performing these modalities are not consistent and varies in different parts of the world [1,25,26]. The observed differences in guidelines some times are due to scarce resources and in some instances are due to socio-cultural factors. For instance while in the USA mammography is routinely offered for every healthy woman above age 40 [23,27], in some poor resource countries there is no possibility of providing such services for women. Evidence for socio-cultural differences in adherent to breast cancer screening programs is also well documented [28-30]. For example, studies found that aboriginal women with different cultural backgrounds living in developed countries attend for regular mammography less than native women [31], or in some countries women hold the believe that if one examines her breast she might develop breast cancer [32,33].

At present there is no a national population-based screening mammography program in Iran, although it is estimated that there are about 200 diagnostic mammography centers in the country (personal communication). If women complain about her breasts usually physicians may ask women to come back with a diagnostic mammogram. The data for diagnostic mammography in Iran varies from 1.3% to 30% [34,35]. Unfortunately the statistics for clinical breast examination does not exist, but it has been estimated that at best 10% of Iranian women living in urban areas might attend for annual clinical breast care [36].

To this end one might conclude that lifestyle modifications and screening behaviors are important issues in breast cancer prevention programs. However, it is important to consider that none of the behaviors discussed could be regarded as the best behavior for breast cancer prevention. Thus, this study aimed to explore Iranian women’s experiences of breast cancer- preventive behaviors, and provide more insight on the topic. It was hoped this piece of work could contribute to the existing literature from a country where lifestyle modification interventions and screening programs are lacking and women live in a cross road of tradition, culture, religion and modernity.

## Methods

### Design and participants

This was a qualitative study. Participants for this study were recruited from a health care center affiliated to Tehran University of Medical Sciences. In order to gain a different perspective we used purposive sampling method to insure that women with varying levels of socioeconomic status, educational level, age, occupation and marital status are present in the study. In addition a team of experts were asked to participate in the study and comment on the topic.

### Data collection

The data were collected using two approaches: focus group discussions, and in-depth interviews with laywomen.

i. Focus group discussions

We conducted two focus group discussions each comprising of eight women in order to explore the issue. Sessions were held in locations that were convenient and accessible for participants. Each session lasted for three and half hours.

ii. Semi structured interviews

Following focus group discussions, in order to get deeper information, 16 semi-structured, open-ended interviews were conducted with laywomen. At the beginning of each interview, the women were asked about demographic characteristics. The interviewer followed a guide designed to encourage women to explore and discuss their knowledge, perception and action about breast cancer. We also asked about social, cultural and environmental factors that might have influenced their behaviors. Then, focusing on each of the expressed behaviors, we asked some questions such as ‘What do you do to prevent breast cancer?’ What are behaviors that might prevent breast cancer? What factors are associated with behaviors that might prevent breast cancer? How do you practice preventive behaviors? Following each interviews women were asked to explain more about what they have said. For instance they were asked ‘What do you mean?’ ‘Explain more’ in order to have deeper understanding of experiences of women. The interviews were carried out at participants’ home or at a public place convenient for the respondent. Each interviews lasted for approximately 1.5–2 hours.

### Data analysis

A content analysis with a conventional approach was carried out to analyze the data and to identify key themes as well as comparing trends and patterns occurred across the various groups. As such the main investigator read the interviews several times and discussed the best approach about coding. During the period of analysis, researcher continually checked similarities and differences in the data and manuscripts, and selected main concepts and formed analytical categories by using comparison of each part of data with other data. We imported the transcripts into the MAXQDA software version 10.

### Ethics

The ethics committee of Tehran University of Medical Sciences approved the study and all participants completed informed written consent.

## Results

In all 24 women aged 30 and over without breast cancer took part in the study. The characteristics of the participants are reported in Table 1. Overall five major themes emerged from the analysis: attitude toward breast cancer and preventive behaviors, stress management, healthy lifestyle, perceived social support and individual and environmental barriers. More information on themes and categories are presented in Table 2. However, in the following section we reflect women’s experiences on major themes.

**Table 1:** Socio demographic characteristics of participants (n = 24)

	**Number**	**Percentage**
**Age group**		
30–34	4	16.66
35–39	9	37.5
40 and above	11	45.84
**Education**		
Primary	4	16.66
Secondary	7	29.16
Higher	13	54.18
**Marital status**		
Single/divorced/widowed	8	33.34
Married	16	66.66
**Occupation**		
Housewife	8	33.34
Employed	16	66.66

**Table 2:** Main themes and categories

**Main themes**	**Categories**
*Attitudes toward breast cancer and preventive behaviors*	
	Beliefs
	Fears and worries
	Individual responsibility
*Stress management*	
	Relaxation
	Positive thinking
	Prayer
*Healthy lifestyle*	
	Having healthy diets
	Sufficient exercise
	Regular assessment of breast health
*Perceived social support*	
	Family and friends
	Media support
	Insurance agencies
*Barriers*	
	Individual barriers
	Environmental constraints

### Attitudes toward breast cancer and preventive behaviors

One of the elicited themes in the present study was attitudes toward breast cancer and preventive behaviors including three different categories namely beliefs, fears and worries, and individual responsibility. Women also expressed different views about breast cancer.

Most participants linked breast cancer to death. Some women believed that even talking about breast cancer might put one at risk of getting breast cancer:

*‘I don’t even want to talk about breast cancer… talking about breast cancer brings bad luck....’* (Participant 14)

Many participants described the negative effects of breast cancer on women’s physical appearance. Some believed that breast cancer is equal to loss of femininity. A woman said:

*‘I hate to hear the word breast cancer. In my view, for a woman, breast cancer is the loss of identity and charm of feminine. I think after that, woman feels unattractive. It is equal with death. It is the end of good life. I am very scared of cancer’* (Participant 2)

A woman indicated that leaving without breasts is meaningless. She said:

*‘I know the breasts are very important to woman. Because of their maternal role, they need to breastfeed their babies. Also women with breast look beautiful. A woman is never complete and beautiful without breast. I would prefer to die than to live without my breasts*’ (Participant 7).

There were mixed beliefs linked with abilities of women about breast cancer-preventive behaviors including healthy lifestyle and screening behaviors: A woman said:

*‘I think if women care for themselves, they must be pay attention to their own breast health by healthy lifestyle, breast check-up and screening’.* (Participant 4)

In contrast a woman stated: *‘I do not go for mammography; I really scare in case it will become breast cancer, I have these things in my mind’.* (Participant 9)

The majority of women expressed fears and worries over their future life. They believed that breast cancer has some social implications such as divorce. They were also worried about their children’s fate. A participant explained that:

*‘I think developing breast cancer might lead to divorce. It might be the end of a married life’* (Participant.11) or it was said that:

*‘The most important thing is my children’s future and happiness. I said to myself I should stay healthy for my children’.* (Participant 6)

Desire to perform preventive behaviors is an important step for prevention of this disease influenced by several factors, including individual responsibility, ability to deal with negative elements of life, effort to maintain normal life, and deal with health barriers. The majority of participants believed that they were able to do preventive behaviors such as regular breast examinations. One participant mentioned that:

‘*Everyone has an important role in own health. If I am not responsible to my health, others cannot help me. I am aware of my abilities. I always try to deal with the negative factors affected my health especially, about an awful disease like breast cancer.’* (Participant.7)

Another participant said:

‘*I think for better and healthier future. I will do everything that has a positive effect on my physical and mental health such as healthy diet, regular exercise and breast exam. Because I love myself, and my health is important to me. I am responsible for my health.’* (Participant.4)

In summary we found that beliefs, fears and worries, and personal responsibility were the most important components of women’s attitudes toward breast cancer.

### Stress management

Since many women believed that stress could cause breast cancer, thus they used different techniques including relaxation, positive thinking and prayer to control their stress and in fact fight against breast cancer. According to one participant:

‘*Yuga helps me to become relax and calm. I believe this makes me strong enough to fight breast cancer*’ (Participant.1)

Another woman said: ‘*I go t*o *meditation classes once a week, because it makes me relaxed and will prevent me from getting breast cancer’* (Participant.4)

Women also expressed other ways to manage their stress to safe guard themselves against breast cancer. For instance the use of prayer was evident in women’s sayings. For many participants, believe in God and prayer, played an important role to prevent cancer. A participant pointed out

*‘I believe that prayer is a source of comfort and composure. Praying and trusting in God get me a peace of mind to care my health. I think it helps me in a spiritual sense and in making me more positive. I believe* that *healing* can *be received from God.’* (Participant. 4)

A positive thinking was believed to be helpful to the final outcome of the breast cancer prevention as expressed by a woman:

*‘It’s very important to have a balance between physical, spiritual, and mental health. This involves working out, eating, believing spirituality, setting life goals, etc.’* (Participant.7)

### Healthy lifestyle

The majority of participants agreed that breast cancer could be prevented by a healthy lifestyle including having healthy diets, doing sufficient exercise, and attending breast check up. Some women made improvements in dietary fat reduction. One of the women mentioned:

*‘I think the best way against breast cancer is healthy lifestyle. I eat healthy foods such as milk, vegetables, low fat, and fruits in my diet. Also, I exercise at least for one hour two or three times weekly’*. (Participant. 3)

Another woman said: *‘I really changed my eating habits and tried to use low-fat foods…’* (Participant. 9)

Some participants put more value on their health and paid more attention to their self-care in order to gain a perfect life. To have clinical breast checkup pointed out by some women:

*‘I went to the doctor for a breast exam. I was so happy, and I forgot all of my worries when the doctor reassured me about my breast health.’* (Participant. 6)

Other participant said:

*‘If I feel something is wrong with my breasts I just go to the doctor, I don’t hesitate going to see a doctor at all even a male doctor’.* (Participant. 11)

Most women mentioned they want living life to the full, thus they care about their breasts. One participant stated:

*‘I realized that I have so many reasons to be healthy and live longer. I care about my breasts so that could enjoy my life.’* (Participant. 1)

### Perceived social support

Both financial and emotional aspects of social support were perceived as fundamental elements of the preventive process for women. Although many participants acknowledged fear and doubts upon hearing the word breast cancer and screening, social support from family and friends served as a source of motivation, enhanced confidence, lessened fears or having someone to encourage them for performing preventive behaviors. One woman said:

*“We’re a happy family. When I go for doing mammography, my husband supports me continuously. He encourages me to do it. He tells: I don’t like that you lose your life due to the breast cancer, I would like to see you safe and healthy.”* (Participant.9)

A few women stated that they received emotional support from friends. They could share their experiences about mammography and BSE. These women felt encouragement for performing preventive behaviors by friends. Peers also provided a means of receiving valuable information regarding breast cancer prevention that they said they did not receive from physicians. A woman pointed out:

*‘I’m willing to do mammography because many of my friends do regularly and they encourage me to do so. Encouragments by friends is very worthwhile…’* (Participant.5)

Also, media (television, films, magazines, and Internet) could positively affect on encouraging women for performing preventive behaviors. It is important to be aware of how women may interact with or be exposed to these types of media. Our understanding of these and our knowledge of where to go for help may prevent a breast cancer. Media can also be used to increase awareness and provide information on breast cancer prevention programs. One woman said that:

*‘I feel more courage getting mammography after watching TV about woman suffering from breast cancer. I know that the mammography is necessary for early detection and prevention of breast cancer*’. (Participant.8)

Another participant said:

*‘I read an article about proper diet and exercise in Internet that is effective for breast cancer prevention. So, I regularly use vegetable, fruit and low-fat diet. That’s what I have been doing’* (Participant.10)

Having health care insurance, and good financial position are some of the effective factors which can encourage women to follow healthy behaviors and also can give them peace of mind. One woman said that

*‘They can offer mammography for free in the government clinics, right?’* (Participant.16)

*“I saw some women that they didn’t receive mammography due to poor financial position and lack of health care insurance. But I did it on time. It is very awful to lose my breasts because of money.”* (Participant.9)

### Individual and environmental barriers

Participants demonstrated that there were many barriers to achieve effective prevention of breast cancer and performing preventive behaviors. These ranged from educational and behavioral aspects to cultural and economic concerns including lack of knowledge and skill, misinformation, doubts, fears, shame, bad experiences and ignorance about risks. Many women reported that they were reluctant to visit doctor, and that were hesitant, or even delayed mammography, because they embarrassed to be examined by a male physicians. They preferred female physicians for breast exams. Although, they was uneasy about breast exams by female physicians. A participant stated that:

*‘I’m willing to do regular breast self-examination, but I don’t know how to do it. I don’t like being seen visiting a male physicians. I feel embarrassed when I have to show my breasts in front of someone especially male doctors…’* (Participant.2)

One woman said that she would never go for a breast exam because she felt so ashamed the last time she went.

*‘I had a breast exam about 3 years ago. When he was doing the breast exam, I felt really ashamed. He did not talk anything when he was doing the breast exams. After the exams, he said that everything is fine. I really do not want to go through the exams (like that) again’* (Participant.4)

One woman mentioned that she didn’t breast exam due to the lack of knowledge about it.

*‘I think, there’s really not enough information out there. My doctor said that you are the second case during thirty years of my medical practice that seeks clinical breast examination’.* (Participant.11)

A few women said that they did not get screening for breast cancer because they believed that it was carcinogenic, and others felt that they were not scared about breast cancer them because they did not have any symptoms or any family history of breast cancer. They had little risk of developing breast cancer. They thought that breast cancer would happen only in older women or those with a family history of breast cancer. A woman said

*‘I have not done mammography because it is carcinogenic. It seems to me that breast cancer is for old women. I don’t think I am likely to get breast cancer. In my family no one has history of breast cancer, so I don’t have any risk of breast cancer’.* (Participant.15)

A woman considered herself as risk free and said:

‘*I am not at all at risk of the breast cancer so why I should* visit *doctor. Nobody in my family has breast cancer. So, I never thought about breast cancer. I don’t think that it would happen to me.*

*I do not want to do it. When I am health, why I make problem for myself. I imagine it is going to hurt me”* (Participant.5)

Also, since there is no national screening program for breast cancer in Iran, some women mentioned that this has negative effect on their behaviors. They stated that the government could make annual mammography for free through cooperation with insurance agencies. Some women proposed free mammogram offered by government at a convenient time. A lady with higher education pointed out:

‘*As far as I know, unfortunately, there is no national screening program of mammography for breast cancer in Iran’*. (Participant.1)

Another woman said, *“In my opinion, the government and ministry of health in cooperation with insurance companies can help women to cover the charge of periodic mammography. They can offer mammography for free in the government clinics, right?”* (Participant.4)

Having financial support had positive psychological effect on participants for attending for annual mammography. In some cases, high cost of mammography was a barrier. One woman mentioned:

*“I’m house wife and my husband has not a good job. Our income is not enough. My doctor recommended doing mammography, but I cannot afford it. If we had enough income I could care of my health much better.” *(Participant.5)

Another participant stated,

*“Although I need to have the breast check-up, I cannot do it because my insurance company does not cover the cost of mammography. I think mammography is very expensive’* (Participant.11)

## Discussion

This study was designed to explore Iranian women’s experiences of breast cancer-preventive behaviors. As a result, several core concepts including attitudes toward breast cancer, preventive behaviors, stress management, healthy lifestyle, perceived social support and individual/environmental barriers emerged. However, one might argue that the analysis did not focus only on preventive behaviors but addressed attitudes, and mammography (which is early detection and screening and prevention). There is evidence that having strong interest and positive attitude to maintain breast health may encourage women for performing preventive action against breast cancer [37]. Thus we believe the emergence of attitude-related concept from the analysis was inevitable.

The findings highlighted that social, cultural and religious attitudes affected breast cancer-preventive behaviors, and there were numerous misconceptions. Many participants stated that they were afraid or scared of the word breast cancer and some believed that even talking about it might cause breast cancer. Similar findings reported by previous studies found that there were lack of knowledge, fear, misconceptions, and fatalistic beliefs among women [38]. The fact that this study’s findings showed that Iranian women believed some myths about the causes of breast cancer were linked to lack of knowledge. It seems that providing ccorrect and timely information might help to overcome these misconceptions and worries about breast cancer.

As same as other studies some women had physical/emotional concerns toward breast cancer [39,40]. They believed that breast cancer is equal to losing their femininity. They believed that breasts are symbol of femininity and sexuality. Such perceptions seem similar among women worldwide. Studies from different women populations in Asia, Africa, Europe and America also reported that women are very concerned with their breasts, as they believe breasts shape up their identity [41-45]. Perhaps those who work in primary health care services might use this as reinforcing factor to encourage women to see their doctors when they feel something is wrong with their breasts.

There have been mixed results about stress and breast cancer. As same as previous studies [46-49], our study showed that many women believed that environmental stress were associated with breast cancer, and they used relaxation, positive thinking and spirituality in order to control their stress. Uncertainty regarding risk of stress on breast cancer may be due to the changing nature of breast cancer research. As a result, stress management not only improves women’s health but also further enhances breast cancer-preventive behaviors.

Most women noted that healthy lifestyle including having healthy diet, exercise and weight control helped them to fight against breast cancer. We thought these are good indications that highlight the fact that women are becoming more alert about factors that jeopardize their life and that they are prepared to change their habits if health care workers recognize such important potentials for behavior changes among women.

Using different levels of emotional, financial, and informational supports motivated women to do preventive behaviors due to increased self-confidence. Previous literature also showed that encouragement by family members, physicians and friends leads to participation in performing preventive behaviors [50-53]. One might argue that breast cancer-preventive behaviors are a socio-ecological subject that needs appropriate informing and social support to promote and to motivate women regarding effective decisions.

According to our findings, lack of knowledge, doubt, fear, shame, hesitancy, busy lifestyle, and fatalism were some barriers to perform preventive behaviors. Similarly, several studies have shown that women did not follow preventive behaviors due to fear of the results, embarrassment, pain, discomfort, and doubt about the efficacy of mammography, high cost of mammography, lack of health care service, and lack of insurance service [54-56]. As same as previous studies [57,58] our findings shown that women believed that risk was greatly influenced by family history, and since there was no breast cancer among their relatives thus they felt they did not need to have a mammogram.

### Limitations

Limitations include the potential that we studied women living in Tehran (the capital) and thus their views may not adequately reflect the views of women living in other settings. Further qualitative and quantitative investigations are needed to examine the experiences and needs of women from other regions and cultures.

## Conclusions

The findings indicated that positive attitudes; individual’s ability to control stress, healthy lifestyle, social support, and fewer barriers are most important parameters that might encourage women to perform breast cancer preventive behaviors.

## Competing interests

The authors declare that they have no competing interests.

## Authors’ contributions

MK was the main investigator and involved in the study design, data collection, analysis and drafting of the manuscript. AM was involved in the study design, data analysis, and providing the final draft. FM contributed to the idea, study design, data analysis and, drafting of the manuscript. AR was involved in the study design, and data analysis. SN and DS participated in the data analysis and, drafting of the manuscript. All authors read and approved the final manuscript.

## Pre-publication history

The pre-publication history for this paper can be accessed here:

http://www.biomedcentral.com/1472-6874/14/41/prepub
